# Supplementary material for: Evidence for the Transcription of a Satellite DNA Widely Found in Frogs
Source: Genes (Basel). 2024 Dec 5;15(12):1572. doi: 10.3390/genes15121572 (PMC11675491; doi:10.3390/genes15121572)
Supplement: Supplementary file 1 [file genes-15-01572-s001.zip › Supplementary Figures_revised_2_Pompeo et al.pdf]

Supplementary figures for:

Evidence for the transcription of a satellite DNA widely found in frogs

Jennifer Nunes Pompeo, Kaleb Pretto Gatto, Diego Baldo, Luciana Bolsoni Lourenço

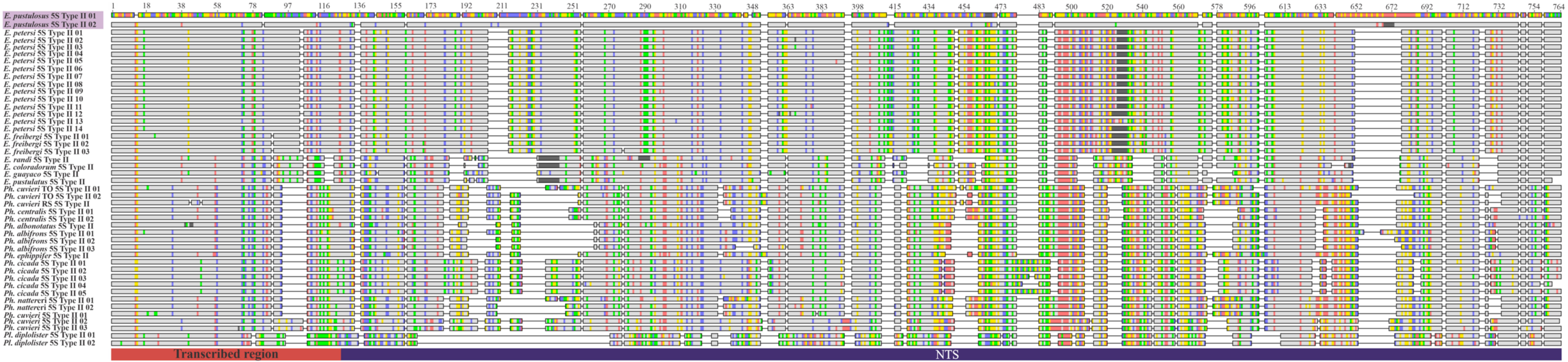

Figure S1. Alignment of all type II 5S rDNA sequences from the subfamily Leiuperinae. The sequences of *E. pustulosus* are highlighted.

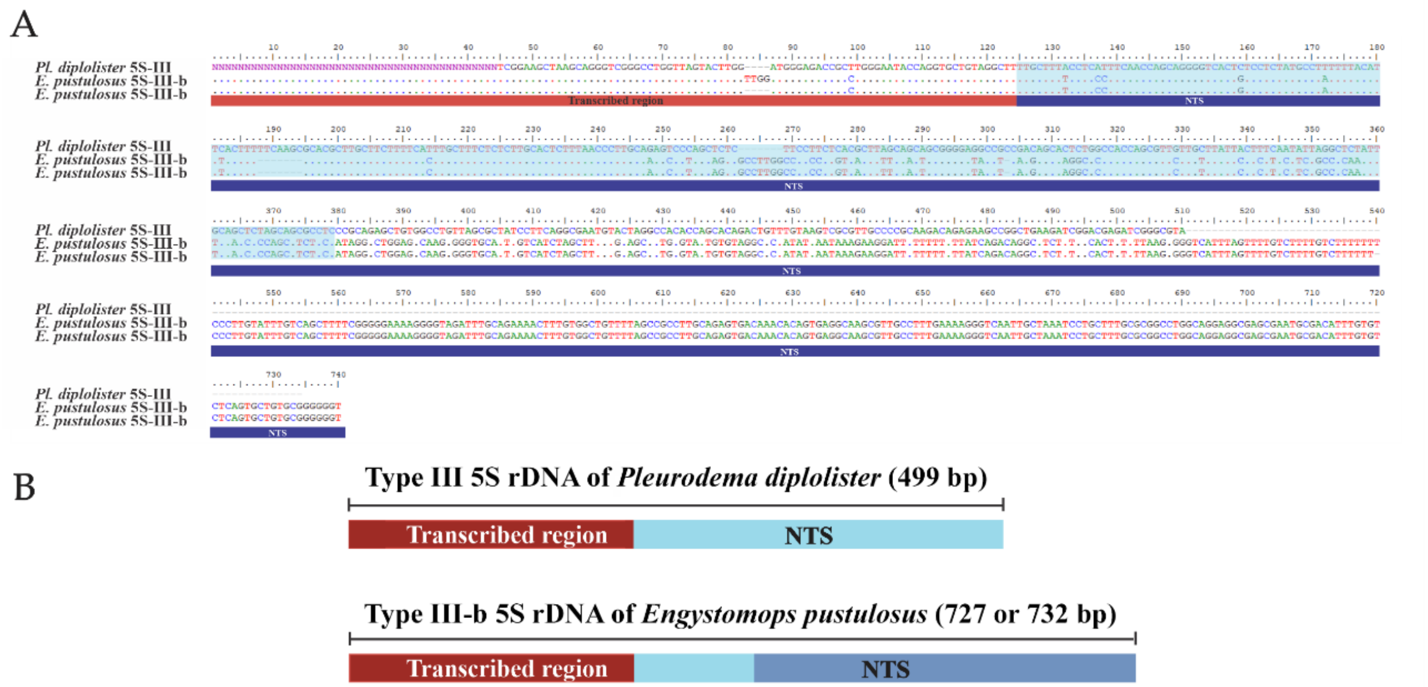

Figure S2. Type III-b 5S rDNA sequence of *E. pustulosus*. A. Alignment of the type III 5S rDNA sequences from *Pl. diplolister* and type III-b 5S rDNA from *E. pustulosus*. The highlighting indicates the 261 bp region of the NTS that shares high similarity between the sequences. B. Schematic representation of the similarity between type III 5S rDNA from *Pl. diplolister* and type III-b 5S rDNA from *E. pustulosus* sequences.

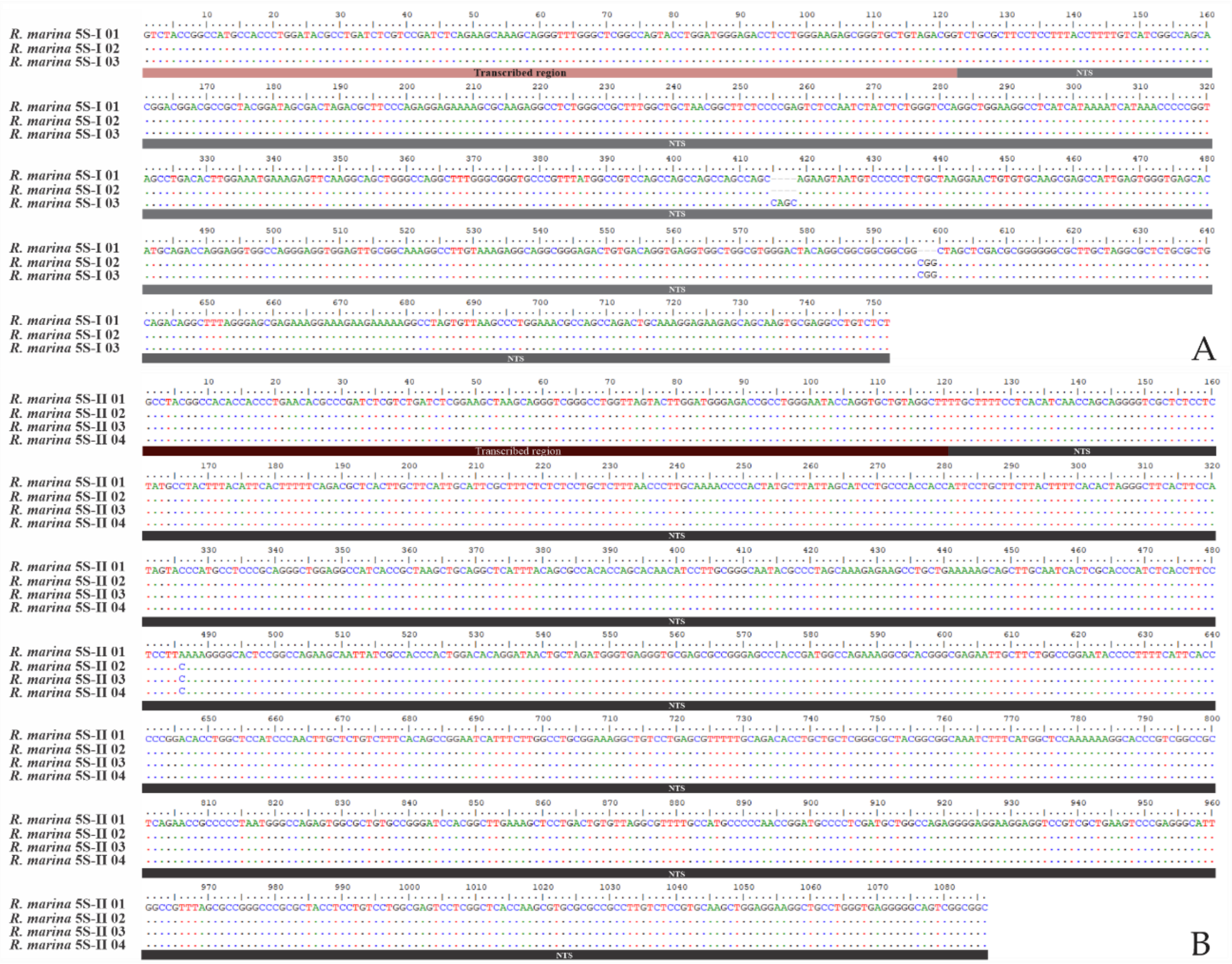

Figure S3. Alignment of the 5S rDNA sequences extracted from the genome assembly of *R. marina*. A. Type I 5S rDNA sequences. B. Type II 5S rDNA sequences.

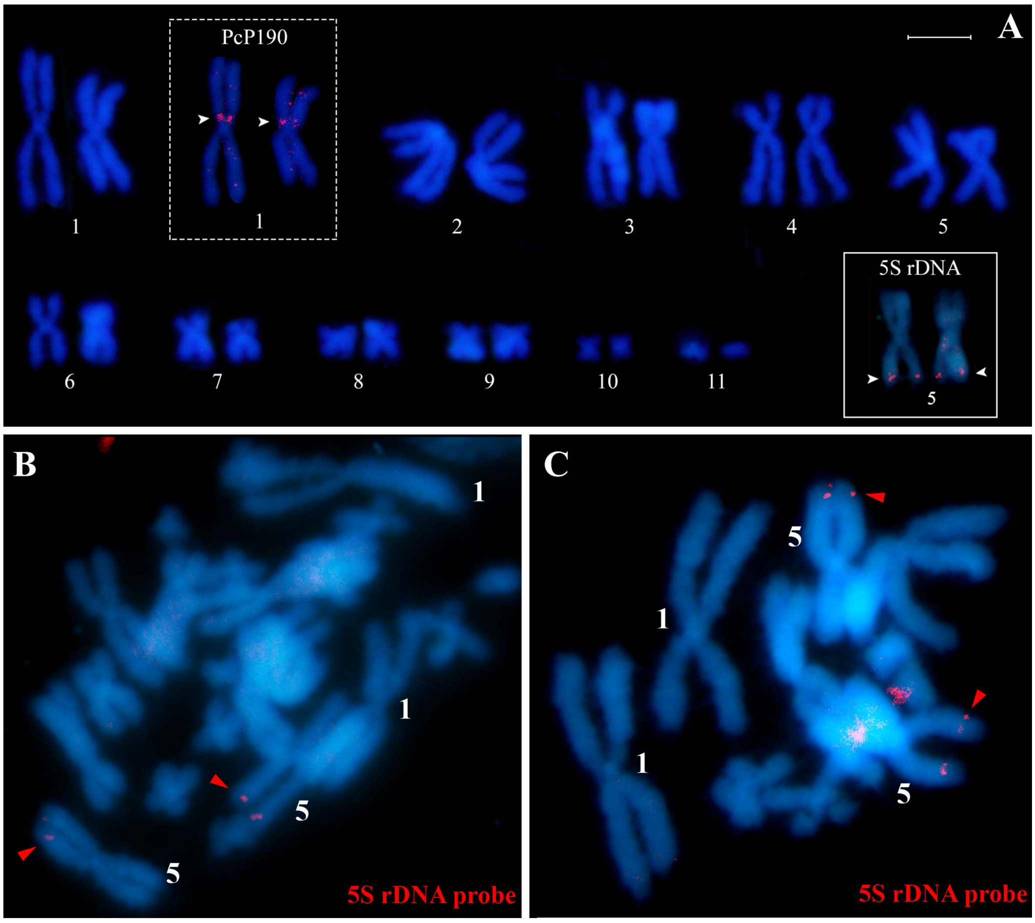

Figure S4. Chromosome mapping of satDNA PcP190 and 5S rDNA in *R. marina*. A. Karyotype of *R. marina* stained with DAPI, and chromosome pairs 1 (the same as in the organized karyotype) and 5 hybridized with a PcP190 probe and a 5S rDNA probe, respectively, shown in dashed line and solid line insets. Note that the PcP190 and 5S rDNA chromosomal clusters do not colocalize. Bar: 5 μm. B-C. Metaphase spreads hybridized with a 5S rDNA probe. Note the probe signals (arrows) in chromosomes 5, while no probe signals are observed in chromosome pair 1.

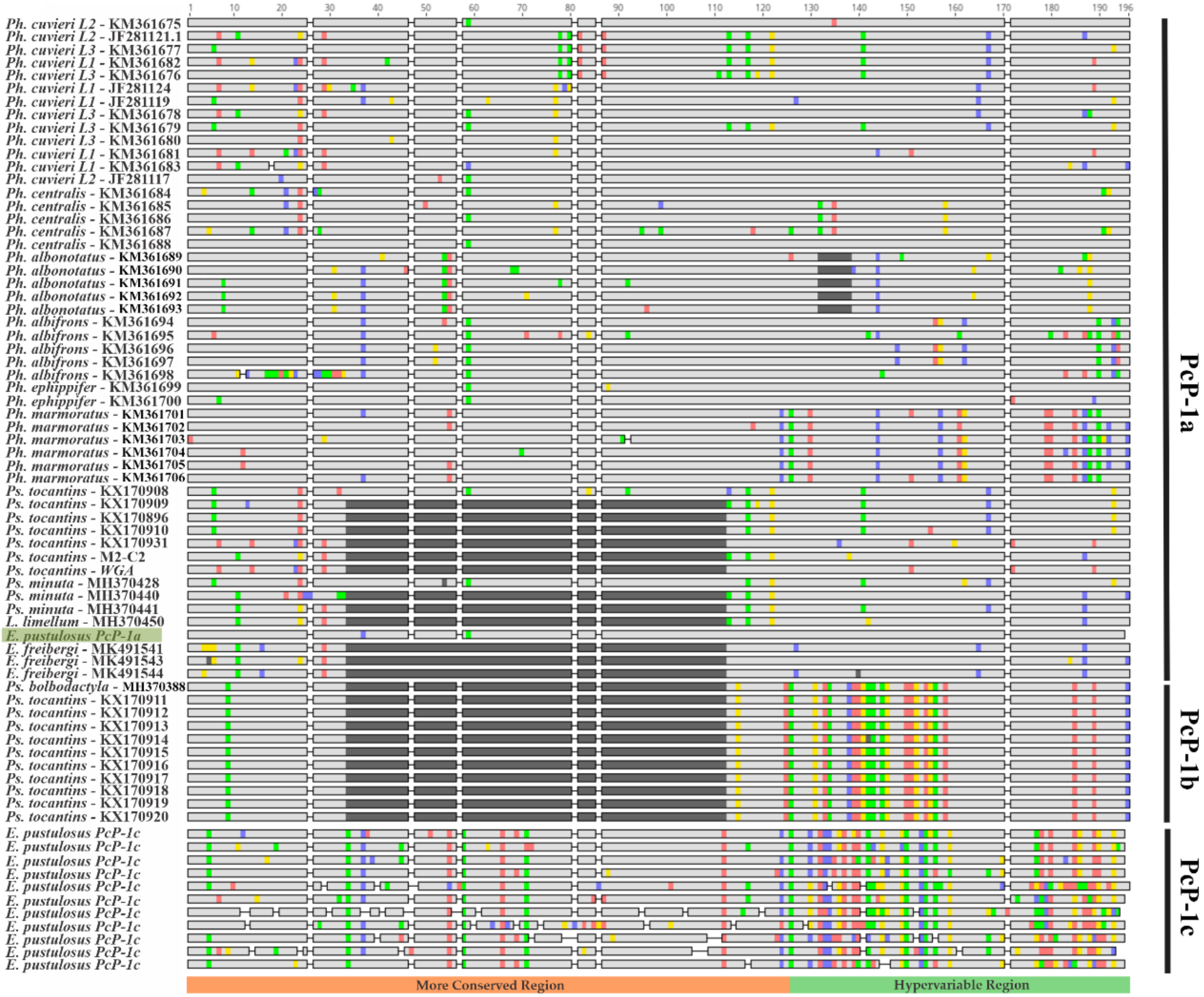

Figure S5. Alignment of all sequences included in Group 1 of the PcP190 satDNA. The PcP-1a sequence from *E. pustulosus* is highlighted. Note that although the HRs of the PcP-1a, PcP-1b, and PcP-1c sequences can be easily distinguished from each other, they can be aligned and share regions of high similarity.

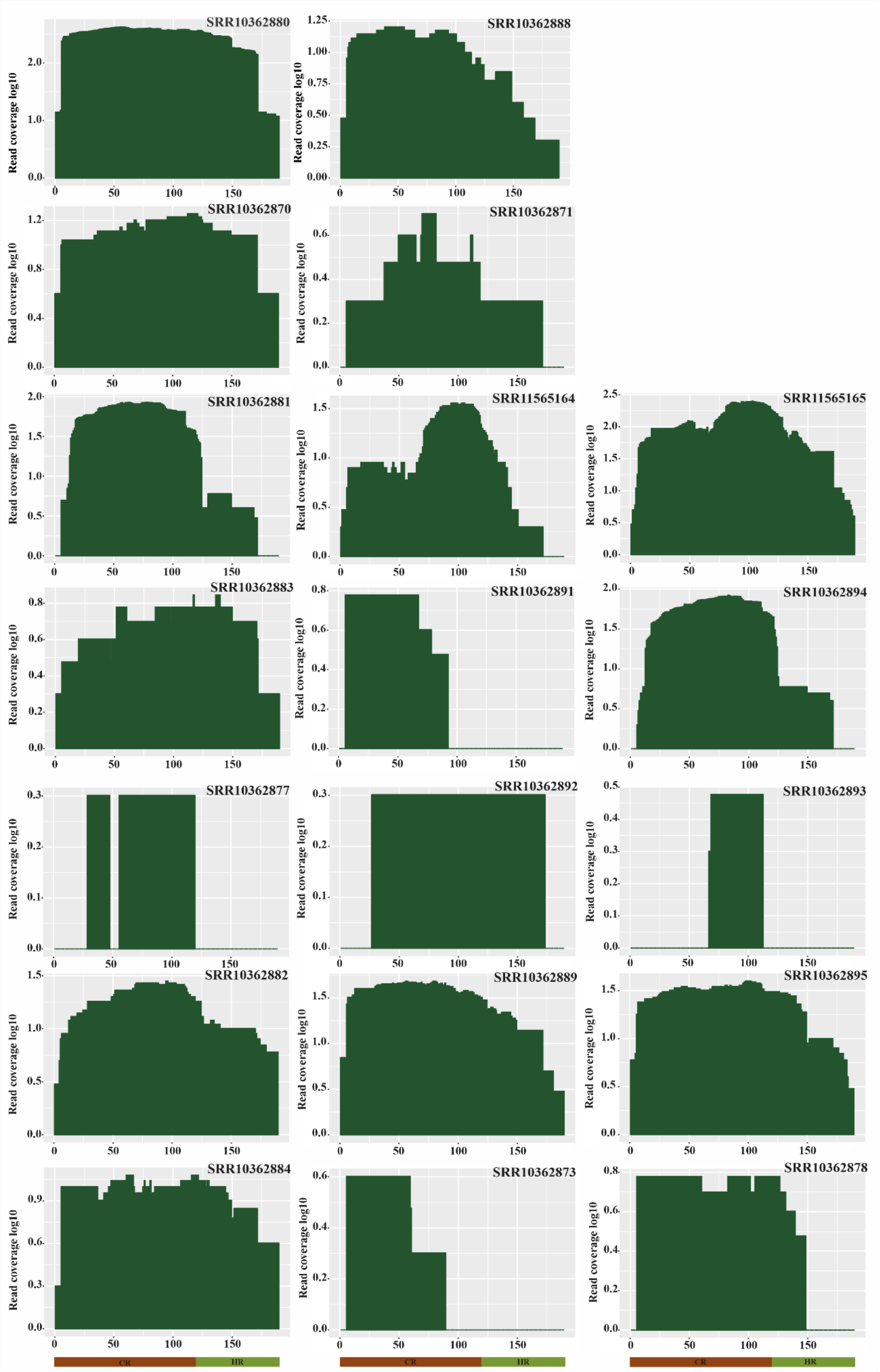

Figure S6. Mapping of reads from RNA-seq libraries of *E. pustulosus* to the consensus sequence of the repetitive unit of the satDNA PcP-1c. The lower bar indicates the extent of the CR (brown) and HR (green) aligned to the plot graphics. The accession numbers of the RNA-seq libraries in the NCBI-SRA database are indicated in each plot. The results for three RNA-seq libraries (SRR10362875, SRR10362886, SRR10362887) are not shown because they had only one read mapped to the PcP sequence (see Supplementary Table 2).

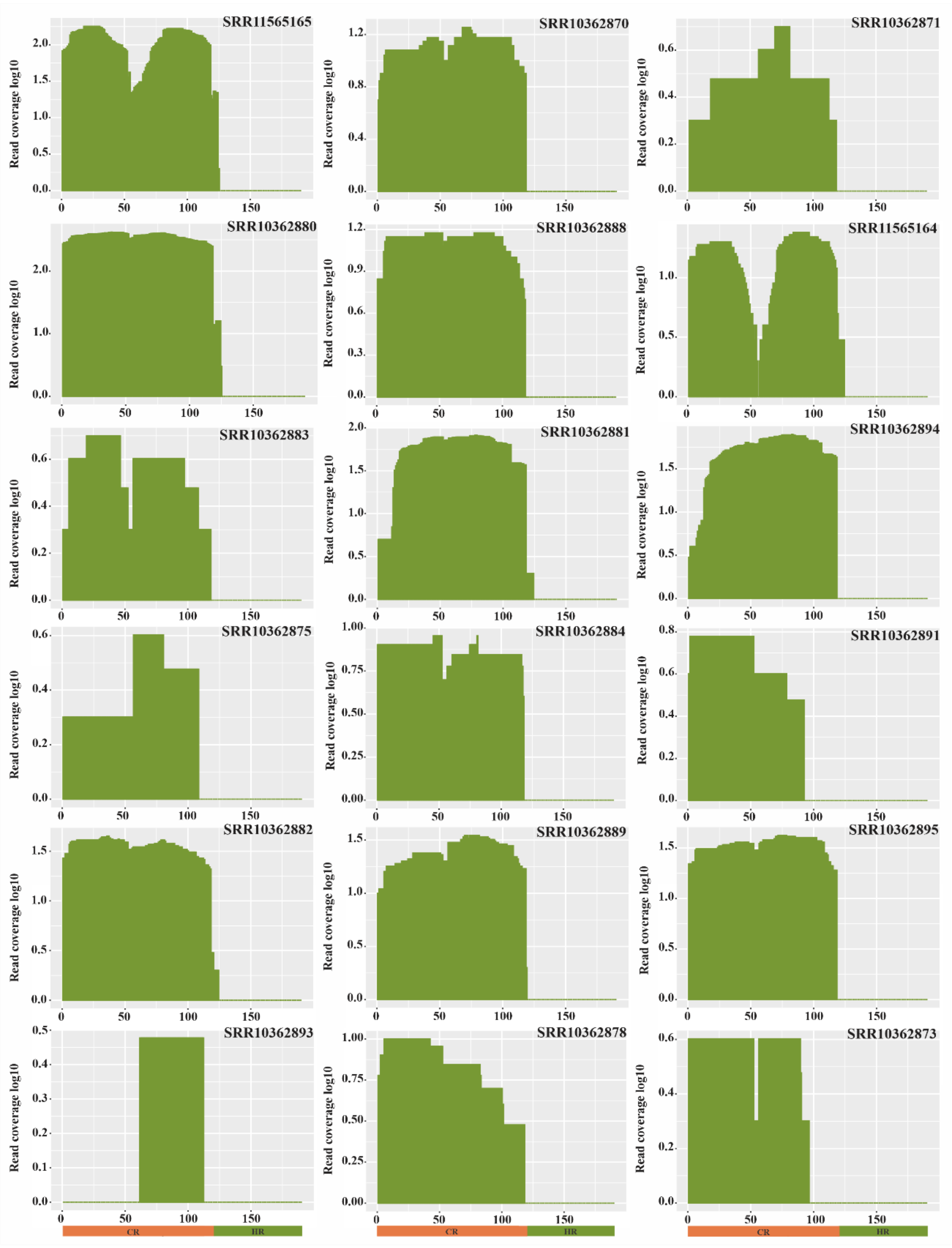

Figure S7. Mapping of reads from RNA-seq libraries of *E. pustulosus* to the consensus sequence of the repetitive unit of the satDNA PcP-1a. The lower bar indicates the extent of the CR (in orange) and HR (in green) aligned correctly to the plot graphics. The accession numbers of the RNA-seq libraries in the NCBI-SRA database are indicated in each plot.

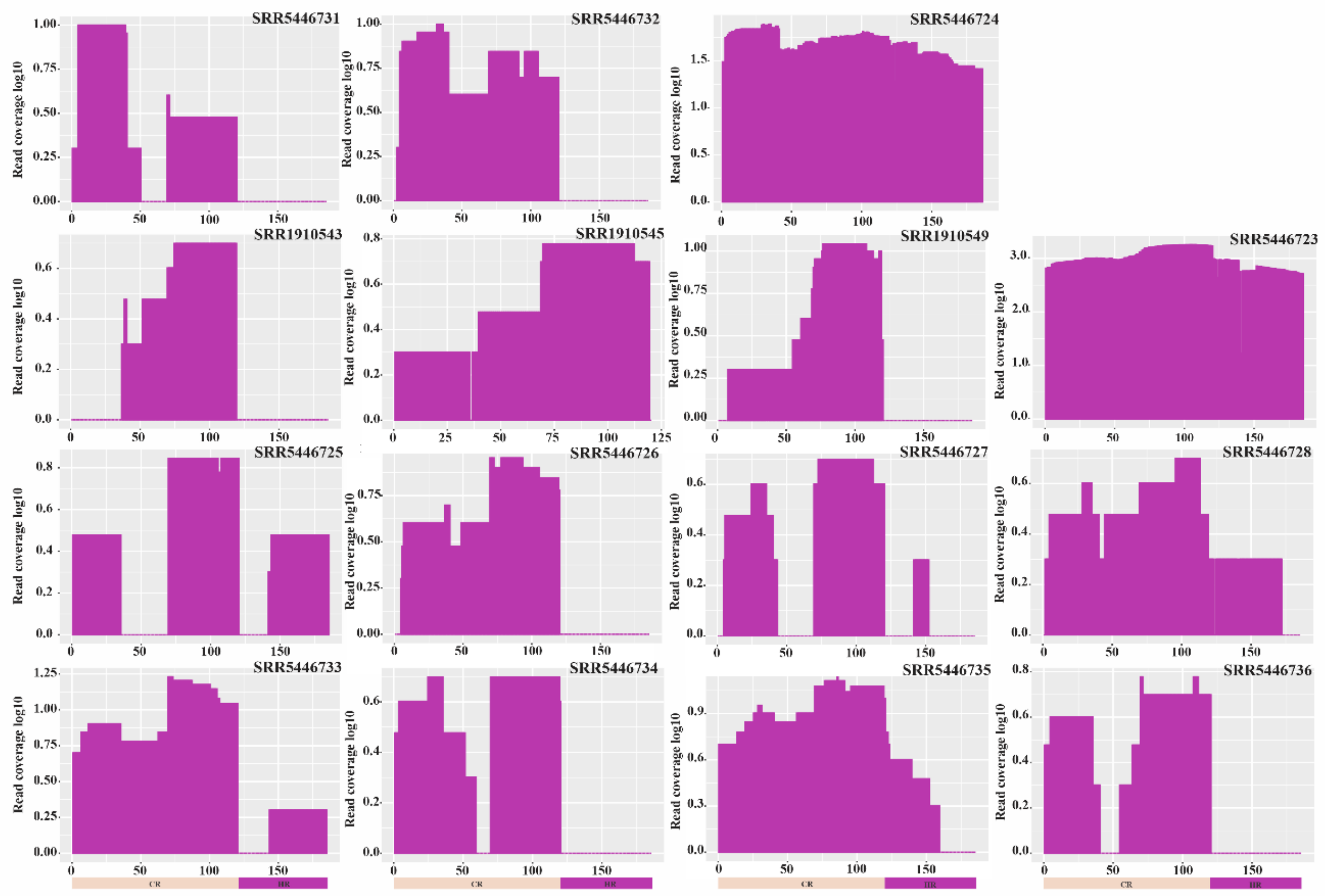

Figure S8. Mapping of reads from RNA-seq libraries of *R. marina* to the consensus sequence of the repetitive unit of the satDNA PcP190 from this species. The lower bar indicates the extent of the CR (in light pink) and HR (in dark pink) aligned correctly to the plot graphics. The accession numbers of the RNA-seq libraries in the NCBI-SRA database are indicated in each plot.
