# Supplementary material for: Evidence for the Transcription of a Satellite DNA Widely Found in Frogs
Source: Genes (Basel). 2024 Dec 5;15(12):1572. doi: 10.3390/genes15121572 (PMC11675491; doi:10.3390/genes15121572)
Supplement: Supplementary file 1 [file genes-15-01572-s001.zip › Supplementary Tables_GENES_Pompeo et al.pdf]

Supplementary data – tables for:

Evidence for the transcription of a satellite DNA widely found in frogs

Jennifer Nunes Pompeo, Kaleb Pretto Gatto, Diego Baldo, Luciana Bolsoni Lourenço

Table S1. PcP190 satDNA and 5S rDNA sequences previously available in public databases and included in our analyses.

Taxon	Sequence	Accession number
**Bufonidae**		
Anaxyrus americanus	5S rDNA	X58365
Rhinella marina	5S rDNA and PcP190	GCA_900303285.1
**Cycloramphidae**		
Cycloramphus bolitoglossus	PcP190	MT920589 - MT920606
**Dendrobatidae**		
Oophaga pumilio	5S rDNA and PcP190	GCA_009801035.1
**Dicroglossidae**		
Nanorana parkeri	5S rDNA	GCA_000935625.1
**Hemiphractidae**		
Gastrotheca riobambae	5S rDNA	M74438
**Hylidae**		
Dendropsophus soaresi	5S rDNA-type I/II	OK634330 - OK634341/ OK634342
Lysapsus limellum	PcP190-type 8	MH370443 - MH370456
Lysapsus limelum	5S rDNA-type II	OK634325
Pseudis bolbodactyla	5S rDNA-type I	OK634324
Pseudis bolbodactyla	PcP190-type 1b	MH370388
Pseudis bolbodactyla	PcP190-type 2	MH370389 - MH370399
Pseudis bolbodactyla	PcP190-type 7	MH370400 - MH370402
Pseudis fusca	5S rDNA-type I	OK634326 - OK634329
Pseudis fusca	PcP190-type 2	MH571141 - MH571149
Pseudis minuta	PcP190-type 3	MH370409 - MH370442
Pseudis paradoxa	PcP190-type 2	MH370403 - MH370407

Pseudis tocantins	5S rDNA-type I/II	KX170899 - KX170903/ KX170904 - KX170907
Pseudis tocantins	PcP190-type 1a	KX170908 - KX170910;
Pseudis tocantins	PcP190-type 1b	KX170911 - KX170920
Pseudis tocantins	PcP190-type 7b	KX17089 - KX170897
Pseudis tocantins	PcP190-type 3	KX170931 - KX170933
Pseudis tocantins	PcP190-type 2	KX170921 - KX170930
Pseudis tocantins	PcP190-type 4	KX170887- KX170889
Pseudis tocantins	PcP190-type 5	KX170890; KX170892; KX170898
Pseudis tocantins	PcP190-type 6	KX170891
Pseudis tocantins	PcP190-type 7a	KX170893 - KX170894
**Leptodactylidae**		
Crossodactylodes sp.	PcP190	MZ905361
Crossodactylus gaudichaudii	PcP190	KM361725 - KM361726
Engystomops “magnus”	Leiuperinae 5S rDNA-type I/II	JF325859 / JF325846 -JF325847
Engystomops coloradorum	Leiuperinae 5S rDNA-type I/II	OK634281 / OK634298
Engystomops freibergi	Leiuperinae 5S rDNA-type I/II	JF325868- JF325870/ JF325843- JF325845
Engystomops freibergi	PcP190-type 1a	MK491541 - MK491544
Engystomops freibergi	PcP190	MK491531 - MK491540
Engystomops guayaco	Leiuperinae 5S rDNA-type II	OK634299
Engystomops petersi	Leiuperinae 5S rDNA-type I/II	GU586283; JF325860- JF325867/ GU586284; JF325848-JF325858
Engystomops pustulatus	Leiuperinae 5S rDNA-type II	OK634300
Engystomops pustulosus	5SrDNA and PcP190	GCA_019512145.1
Leptodactylus latrans	PcP190	KM361718 - KM361724
Physalaemus albifrons	Leiuperinae 5S rDNA-type I/II	OK634282 - OK634283/ OK634307 - OK634309
Physalaemus albifrons	PcP190-type 1a	KM361694 - KM361698
Physalaemus albonotatus	Leiuperinae 5S rDNA-type I/II	OK634284 - OK634284/ OK634306
Physalaemus albonotatus	PcP190-type 1a	KM361689 - KM361693
Physalaemus centralis	Leiuperinae 5S rDNA-type I/II	OK634288/ OK634304
Physalaemus centralis	PcP190-type 1a	KM361684 - KM361688

Physalaemus cicada	Leiuperinae 5S rDNA-type I/II	OK634289/ OK634311- OK634315
Physalaemus cuvieri	Leiuperinae 5S rDNA-type I/II	JF281126 - JF281131; JF281132 - JF281134
Physalaemus cuvieri	PcP190-type 1a	KM361675 - KM361683; JF281117; JF281119; JF281121; JF281124
Physalaemus cuvieri L1	Leiuperinae 5S rDNA-type I	OK634291
Physalaemus cuvieri L2	Leiuperinae 5S rDNA-type II	OK634301
Physalaemus cuvieri L3	Leiuperinae 5S rDNA-type I/II/IV	OK634290; OK634302-03; OK634321-22
Physalaemus ephippifer	Leiuperinae 5S rDNA-type I/II	OK634292 - OK634293/ OK634310
Physalaemus ephippifer	PcP190-type 1a	KM361699 - KM361700
Physalaemus kroyeri	Leiuperinae 5S rDNA-type I/IV	OK634294/ OK634323
Physalaemus marmoratus	PcP190-type 1a	KM361701 - KM361706
Physalaemus nattereri	Leiuperinae 5S rDNA-type I/II	OK634295/ OK634316 - OK634317
Pleurodema diplolister	Leiuperinae 5S rDNA-type I/II/III	OK634296/ OK634318 - OK634319/ OK634320
**Megophryidae**		
Leptobrachium leishanense	5S rDNA	GCA_009667805.1
**Odontophrynidae**		
Proceratophrys boiei	PcP190	MN475838
**Pipidae**		
Xenopus borealis	Oocyte 5S rDNA	V01426; V01425
Xenopus borealis	Somatic 5S rDNA	K01537; K01374
Xenopus laevis	Oocyte 5S rDNA	M10027; M10635; M10850; M10676; J01010; X05089; M63899; J01012; M30904; M35055; M35176; K02695
Xenopus laevis	Somatic 5S rDNA	J01009; X12622; J01899; M35175; M35055
Xenopus tropicalis	Somatic 5S rDNA	NR033271
Xenopus tropicalis	Oocyte 5S rDNA	X12624; X12623; NR_033270
**Pyxicephalidae**		
Pyxicephalus adspersus	5S rDNA	GCA_004786255.1
**Ranidae**		
Amolops mantzorum	5S rDNA-type I/II	KX913750; KX913751; KX913753- KX913758; KX913760- KX913765/ KX913766- KX913778
Aquarana catesbeiana	5S rDNA	X58367

Lithobates pipiens	5S rDNA	X58368
Pelophylax lessonae	5S rDNA	FJ572051
Pelophylax ridibundus	5S rDNA	FJ572052
Rana temporaria	5S rDNA	GCA_009802015.1
**Scaphiopodidae**		
Spea multiplicata	5S rDNA	GCA_009364415.1

Table S2. Mapping of reads of the RNA-seq libraries from *Engystomops pustulosus* to the 5S rDNA sequences of this species.

**SRA accession number**	**Tissue**	**Total number of reads mapped onto type II 5S rDNA of *E. pustulosus***	**Total number of reads mapped onto type III-b 5S rDNA of *E. pustulosus***
SRR10362882	Brain	9	259
SRR10362889	Brain	20	355
SRR10362895	Brain	7	394
SRR10362879	Heart	0	23
SRR10362893	Heart	8	46
SRR10362876	Liver	0	18
SRR10362886	Liver	0	24
SRR10362878	Intestine	2	58
SRR10362887	Intestine	0	40
SRR10362872	Larynx	5	210
SRR10362877	Larynx	0	42
SRR10362892	Larynx	2	183
SRR10362880	Eyes	13	183
SRR10362888	Eyes	49	211
SRR10362883	Dorsal Skin	0	168
SRR10362891	Dorsal Skin	96	177
SRR10362873	Ventral skin	2	92
SRR10362890	Ventral skin	81	157
SRR10362875	Lungs	0	64
SRR10362884	Lungs	37	167
SRR11565164	Hippocampus	12	2980
SRR11565165	Hippocampus	17	17,160
SRR10362870	Tadpole	1	114
SRR10362871	Tadpole	0	110
SRR10362881	Eggs	28	109
SRR10362894	Eggs	24	46

Table S3. Mapping of reads of the RNA-seq libraries from *Rhinella marina* to the type I and type II 5S rDNA of this species.

SRA accession number	Tissue	Total number of reads mapped onto type I 5S rDNA of *R. marina*	Total number of reads mapped onto type II 5S rDNA of *R. marina*
SRR5446729	Spleen	0	71
SRR5446730	Spleen	0	11
SRR5446731	Spleen	0	17
SRR5446732	Spleen	0	4
SRR5446733	Brain	0	7
SRR5446734	Brain	0	8
SRR5446735	Brain	0	1
SRR5446736	Brain	0	31
SRR1910534	Muscle	0	6
SRR1910543	Muscle	0	12
SRR1910545	Muscle	0	4
SRR1910549	Muscle	0	0
SRR5446724	Ovaries	2	14
SRR5446723	Testis	66	237
SRR5446725	Tadpole	0	4
SRR5446726	Tadpole	0	0
SRR5446727	Tadpole	0	18
SRR5446728	Tadpole	0	3

Table S4. Mapping of reads of the RNA-seq libraries from *Engystomops pustulosus* to a sequence of PcP190-type c of the same species. For each library, SRA accession number, the number of paired-end (PE) reads, and the tissue and sex (F: female; M: male) of the specimen used to libray construction are indicated. The number of single reads mapped to any region of the PcP-1c sequence and the number of single reads mapped to the conserved region (CR) or the hypervariable region (HR) of this PcP-1c sequence are shown. Note that a single read may map to both CR and HR. The number of fragments that were mapped to the PcP-1c sequence is shown and refers to the number of single reads and PE reads. When PE reads were mapped, only 1 fragment was counted. FPKM: fragments per kilobase of transcript per million mapped reads.

SRA accession number	Number of PE reads	Tissue	Sex	Total number of reads mapped to the PcP-1c	Number of reads mapped to the CR	Number of reads mapped to the HR	Fragments mapped to PcP-1c	FPKM
SRR10362882	14784557	Brain	F	65	59	30	36	12.28
SRR10362889	39972146	Brain	F	31	30	15	19	2.51
SRR10362895	14990273	Brain	F	49	48	32	30	10.58
SRR10362879	11456574	Heart	F	0	0	0	0	0
SRR10362893	10219273	Heart	F	3	3	0	3	1.55
SRR10362876	12880961	Liver	F	0	0	0	0	0
SRR10362886	7629615	Liver	F	1	1	1	1	0.69
SRR10362878	22871643	Intestine	F	9	9	6	5	1.15
SRR10362887	12276818	Intestine	F	1	1	0	1	0.43
SRR10362872	26464434	Larynx	M	0	0	0	0	0
SRR10362877	9315605	Larynx	F	2	2	0	2	1.13
SRR10362892	18889423	Larynx	F	2	2	2	1	0.28
SRR10362880	12717851	Eyes	F	599	565	413	320	133.12
SRR10362888	12381752	Eyes	F	20	18	9	10	4.27
SRR10362883	18862395	Dorsal Skin	F	11	9	7	7	1.96
SRR10362891	13346541	Dorsal Skin	F	6	6	1	3	1.18
SRR10362873	19580626	Ventral skin	F	5	4	1	3	0.81
SRR10362890	18584980	Ventral skin	F	0	0	0	0	0
SRR10362875	8643746	Lungs	F	1	1	0	1	0,61
SRR10362884	13041544	Lungs	F	21	20	10	11	4.46
SRR11565164	28772029	Hippocampus	F	52	50	17	44	8.09
SRR11565165	24388173	Hippocampus	M	436	393	244	346	75.06
SRR10362870	18749949	Tadpole	-	25	25	16	15	4.23
SRR10362871	15071236	Tadpole	-	6	5	2	5	4.23
SRR10362881	20616454	Eggs	-	94	92	24	64	16.42
SRR10362894	9058124	Eggs	-	86	86	6	55	32.12

Table S5. Mapping of reads of the RNA-seq libraries from *Engystomops pustulosus* to a sequence of PcP190-type 1a of the same species. For each library, SRA accession number, the number of paired-end (PE) reads, and the tissue and sex (F: female; M: male) of the specimen used to library construction are indicated. The number of single reads mapped to any region of the PcP-1a sequence and the number of single reads mapped to the conserved region (CR) or the hypervariable region (HR) of this PcP-1a sequence are shown. Note that a single read may map to both CR and HR. The number of fragments that were mapped to the PcP-1a sequence is shown and refers to the number of single reads and PE reads. When PE reads were mapped, only one fragment was counted. FPKM: fragments per kilobase of transcript per million mapped reads.

SRA accession number	Number of PE reads	Tissue	Sex	Total number of reads mapped to PcP-1a	Number of reads mapped to the CR	Number of reads mapped to the HR	Fragments mapped to PcP-1a	FPKM
SRR10362882	14784557	Brain	F	54	54	0	30	10.67
SRR10362889	39972146	Brain	F	39	39	0	24	3.16
SRR10362895	14990273	Brain	F	52	52	0	35	12.28
SRR10362879	11456574	Heart	F	0	0	0	0	0
SRR10362893	10219273	Heart	F	3	3	0	2	1.03
SRR10362876	12880961	Liver	F	0	0	0	0	0
SRR10362886	7629615	Liver	F	1	1	0	1	0.68
SRR10362878	22871643	Intestine	F	10	10	0	5	1.15
SRR10362887	12276818	Intestine	F	1	1	0	1	0,42
SRR10362872	26464434	Larynx	M	0	0	0	0	0
SRR10362877	9315605	Larynx	F	2	2	0	2	1.12
SRR10362892	18889423	Larynx	F	1	1	0	1	0.27
SRR10362880	12717851	Eyes	F	526	526	0	289	119.5
SRR10362888	12381752	Eyes	F	18	18	0	11	4.67
SRR10362883	18862395	Dorsal Skin	F	7	7	0	4	1.11
SRR10362891	13346541	Dorsal Skin	F	6	6	0	3	1.18
SRR10362873	19580626	Ventral skin	F	6	6	0	3	0.80
SRR10362890	18584980	Ventral skin	F	0	0	0	0	0
SRR10362875	8643746	Lungs	F	4	4	0	2	1.21
SRR10362884	13041544	Lungs	F	13	13	0	8	3,22
SRR11565164	28772029	Hippocampus	F	44	44	0	36	6.58
SRR11565165	24388173	Hippocampus	M	346	346	0	289	62.36
SRR10362870	18749949	Tadpole	-	22	22	0	15	4.21
SRR10362871	15071236	Tadpole	-	5	5	0	4	1.39
SRR10362881	20616454	Eggs	-	90	90	0	63	16.08
SRR10362894	9058124	Eggs	-	82	82	0	54	31.37

Table S6. Mapping of reads of the RNA-seq libraries from *Rhinella marina* to a sequence of PcP190 of the same species. For each library, SRA accession number, the number of PE reads, and the tissue and sex (F: female; M: male) of the specimen used to its construction are indicated. The number of single reads mapped to any region of the PcP-1c sequence and the number of single reads mapped to the conserved region (CR) or the hypervariable region (HR) of this PcP-1c sequence are shown. Note that a single read may map to both CR and HR. The number of fragments that were mapped to the PcP-1c sequence is shown and refers to the number of single reads and PE reads. When PE reads were mapped, only one fragment was counted. Note the great number of reads from the testis library that was mapped (highlighted in blue). FPKM: fragments per kilobase of transcript per million mapped reads.

SRA accession number	Number of reads	Tissue	Sex	Total number of reads mapped to PcP	Number of reads mapped to the CR	Number of reads mapped to the HR	Fragments mapped to PcP	FPKM
SRR5446729	11804387	Spleen	F	4	4	0	3	1.37
SRR5446730	12327484	Spleen	F	0	0	0	0	0
SRR5446731	12479474	Spleen	F	13	13	0	8	3.46
SRR5446732	11892208	Spleen	F	13	13	0	9	4.09
SRR5446733	11153836	Brain	F	21	19	2	13	6.3
SRR5446734	12408489	Brain	F	10	9	1	6	6.21
SRR5446735	13862169	Brain	F	16	15	4	9	3.5
SRR5446736	11973029	Brain	F	10	10	1	8	3.61
SRR1910534	46906262	Muscle	F	3	2	1	2	0.23
SRR1910543	44098969	Muscle	F	6	6	0	5	0.61
SRR1910545	48797868	Muscle	F	9	8	1	6	0.66
SRR1910549	39978543	Muscle	F	13	12	1	12	1.62
SRR5446724	217050748	Ovary	F	138	129	54	92	2.29
SRR5446723	205232429	Testis	M	2,793	2,070	1,148	1,876	49.51
SRR5446725	12827175	Tadpole	-	13	11	3	9	3.79
SRR5446726	11599984	Tadpole	-	12	12	1	7	3.26
SRR5446727	12260961	Tadpole	-	9	8	2	7	3.08
SRR5446728	13212386	Tadpole	-	7	5	2	7	2.86
